# Synergistic influence of education and marriage on the risk for cognition loss among the older people in China

**DOI:** 10.1002/alz.083868

**Published:** 2025-01-09

**Authors:** Ning Sun

**Affiliations:** ^1^ Ningbo College of Health Sciences, Ningbo, zhejiang China

## Abstract

**Background:**

The study was aim to prove that both rationality and emotion are indispensable for older people to maintain their ability to live independently during the twilight of their lives.

**Method:**

The resilience of older people to dementia were investigated by considering the interactions between educational levels and marriage status. Four sociodemographic variables (age, sex, educational level, and marital status) were collected from 1177 older Chinese participants, whose mini‐mental state examination scores (MMSE scores) were measured.

**Result:**

A lower educational level coupled with being widowed caused a greater risk for severe cognitive impairment (relative risk [RR] 1.48; 95% confidence interval [CI] 1.20‐1.82; *p* < 0.001) for high‐aged older participants (age range: ≥80) than for their low‐aged counterparts (age range: ≥60 and <80). In contrast, a higher educational level coupled with being married leveled this age‐related risk for cognitive loss (RR 0.91; 95% CI 0.65‐1.27; *p* = 0.62).

**Conclusion:**

Further findings suggest that the synergistic influence of education and marriage was observed only among high‐aged older people. Being both well‐educated and married is associated with a delayed cognitive function for older people. However, longevity is a prerequisite for realizing this benefit.

